# NF-κB1 Rs28362491 Mutant Allele Frequencies along the Silk Road and Beyond

**Published:** 2018-03

**Authors:** Safoora PORDEL, Kazem NEMATI, Mohammad Hossein KARIMI, Mehrnoosh DOROUDCHI

**Affiliations:** 1. Dept. of Immunology, School of Medicine, Shiraz University of Medical Sciences, Shiraz, Iran; 2. Shiraz Blood Transfusion Center, Shiraz, Iran

**Keywords:** NF-*κ*B1, Polymorphism, Normal population, PCR–RFLP, Iran

## Abstract

**Background:**

In the human evolutionary history, Single Nucleotide Polymorphism (SNP) frequencies are valuable in terms of finding connections between different populations. Due to the pronounced role of the immune system in combating pathogens and environmental stressors, polymorphisms in the immune genes are subject to selection pressure of the diseases as well. The functional polymorphisms in NF-κB1 promoter (-94 ins/del) are associated with different diseases; therefore, we aimed to establish the frequencies of NF-κB1 rs28362491 alleles in a population of Southwestern Iranians in comparison with the world populations.

**Methods:**

We assessed the polymorphism of -94 ATTG ins/del (rs28362491) in 201 Iranian healthy blood donors from Fars Province, central Iran in a one year period between 2015 and 2016 by PCR-RFLP method using DNA extracted from peripheral blood mononuclear cells.

**Results:**

The frequency of ins/ins homozygote genotype was found to be 46.97%. The frequency of heterozygote individuals was 42.42% and the percentage of del/del homozygote genotype was 10.61%. We observed a genetic similarity based on the genotype frequencies of NF-κB1 -94 ins/del ATTG polymorphism between our sample of Iranians with American Jewish, Turkish, American non-Jewish, Chinese-Uyghurs and Germans.

**Conclusion:**

The results confirmed genetic interrelation of Iranians with some ancient neighbors and their admixture with countries along the Silk Road. We suggest that mapping the distribution of NF-κB1-94 ATTG ins/del along with HLA genes may help to better define the relations between human populations and design population-specific vaccines for pathogens with a high rate of variation.

## Introduction

NF-κB is a family with several transcription factors which regulate expression of many genes with significant roles in inflammatory and immunologic responses, angiogenesis, apoptosis, differentiation, invasion, cell proliferation and adhesion (1, 2). In mammals, NF-κB family has five members including Rel A (p65), Rel B, Rel (C-Rel), p105 (NF-κB1), and p100 (NFκB2). In addition, there exist p52 and p50 proteins that are processed forms of p100 and p105, respectively. Although many dimeric forms of NF-κB exist, the heterodimer of Rel A (p65) and p105 (p50) is the main form of NFκB molecule (3, 4). The Relations between NFκB activation and the inflammations associated with Asthma, Rheumatoid Arthritis (RA), Septic Shock, Diabetes, AIDS, Stroke, Atherosclerosis, Cancer, Infertility, and Endometriosis are shown (5–7). Owing to the great role of the immune system in combating pathogens and environmental stressors during evolution, polymorphisms in the immune genes are widely used to track the disease susceptibility as well as the history of human populations (8, 9). HLA alleles, for example, are in the heart of immune activation against pathogens; therefore, there are compelling associations between HLA alleles and different diseases (10). The same set of genes are also used to find the footprints of human evolution in the genome (8). Polymorphisms in Toll-like receptors, cytokines and signaling molecule have also been used to decipher the interrelation of human populations and ethnic similarities (11, 12).

Considering the importance of NFκB in the activation of the immune system, one may ask to what extent polymorphisms of this gene may have contributed to the genetic make-up of the current human populations. Several single nucleotide polymorphisms (SNPs) of NFκB1 gene have been reported (13). One of the functional polymorphisms in NFκB1 promoter (-94 ins/del) is shown to be associated with different diseases (7, 14–17). Deletion of 4 base pairs in NFκB1 promoter causes destruction of transcription factor binding site and results in a lower promoter activity and thereby reduction of p50 (p105) expression. P50 homodimers have anti-inflammatory effect while p50/p65 heterodimers have inflammatory effect (18). -94 NFκB AATG2 (ins) frequency is higher in healthy individuals than patient with Aneurysm (14), CAD, ventricular remodeling and impaired LV function (15,16). The same allele increases the risk of colorectal and non-small cell lung cancers (17–19) but not ovarian cancer (20).

Association of NFκB with autoimmune diseases has been inconsistent based on the disease and/or the ethnicity of the population (12, 13). In a Turkish population of patients with Behcet Disease (BD), the ins/ins genotype increased the risk of ocular involvement while heterozygous genotype was protective (21). While in patients with Systemic Lupus Erythematous (SLE) in China heterozygous del/ins genotype decreased the risk of the disease (22). Ulcerative Colitis (UC) was associated with NF-κB1 Del allele in Dutch people (18), but not in British, German and Spanish populations (23–26). These discordant results could be due to the differences in pre-disposing environmental factors for UC in different populations (27).

While polymorphisms are valuable tools for the study of disease associations, they can also be used in tracing human history and studying the effect of natural selection in human populations (11, 28). Through impact on survival and reproductive ability, natural selection is one of the mechanisms that created the divergent distribution of allele frequencies between different human populations (29). Despite different ethnic and genetic backgrounds in two populations, equal pressure, for example plague infection, caused similar evolution in Toll-like receptors (11). A polymorphism in TNF-α, which affects its binding to both NF-κB p65-p50 and p50-p50 dimers, is reported to affect the susceptibility to severe RA and is subject to evolutionary selection pressure (30). An SNP in the regulatory region of CCR5 gene leads to loss of binding of NF-κB transcription factor and is common in different populations (31). NF-κB is known to regulate the expression of cytokines and their receptors and play a central role in the induction of inflammation (32). Therefore, polymorphisms that affect its function and alter its binding affinity to regulatory regions of target genes may be a serious selection pressure that results from host-parasite interrelationship and might define ultimate pathogenic consequences (31).

So far, two studies have investigated the association of this polymorphism with diseases in Iranian population. In one study, a relationship between this polymorphism and breast cancer was found (33), while in another study no association with multiple sclerosis was observed (34).

## Materials and Methods

### Study population

Healthy individuals were recruited from healthy blood donors in Shiraz, Fars Province, central Iran between 2015 and 2016. All participants gave informed consent. Clinical Research Ethics Committee of Shiraz University of Medical Sciences, Shiraz, Iran, approved this descriptive population genetics study.

### Blood samples and DNA extraction

Six ml venous blood was collected from all subjects in tubes containing EDTA as anticoagulant. DNA extraction was performed by salting out methods described previously (35). DNA concentration and protein contamination were determined by means of spectrophotometerat 260 and 280 wavelengths. The DNA samples standardized to 0.3 μg/μL concentration.

### Genotyping

**Fig. 1:**
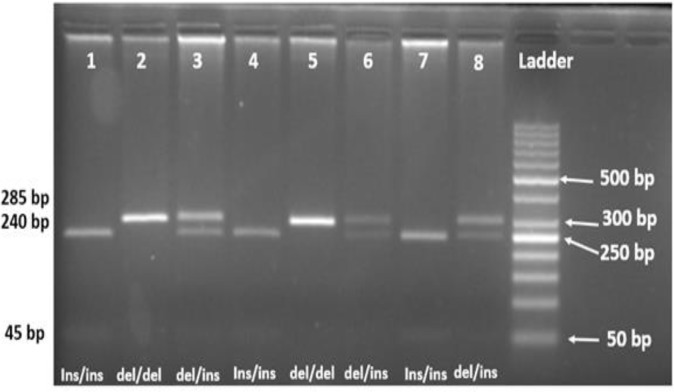
The RFLP products of -94NFκB ins/del ATTG are shown

NF-KB gene is a highly polymorphic gene with several SNPs scattered in the intronic and promoter areas of the gene. We studied -94 ATTG ins/del (rs28362491) polymorphism in the promoter of the NF-KB gene by PCR-RFLP method. PCR reaction was performed in a 15 μl total reaction volume containing 200 μM of each dNTPs, 30 ng genomic DNA, 2 mM of MgCl_2_, 10X PCR Buffer, 1 U Taq DNA polymerase and 1 μM of each primer (10 PM concentration). Then the restriction enzyme was added to the PCR products and incubated at 37 °C overnight in a dry block. The structures of primers, and required restriction enzyme (PflMI) are shown in Table 1. The cleaved product was run on a 3.5 agarose gel (Invitrogen, England) containing 2.5 μl safe stain and genotypes were analyzed by using a UV transilluminator at 254 nm (Fig. 1).

**Table 1:** The structures of primers and required restriction enzyme, as well as length of PCR product is presented

***Polymorphism***	***Primer***	***Length of PCR product (bp)***	***Restriction enzyme***
NF-κB1-94 ins/del ATTG	Forward 5′-TGGGCACAAGTCGTTTATGA-3′ Reverse 5′-CTGGAGCCGGTAGGGAAG-3′	285	Van91I (PflMI)

## Results

We studied 201 healthy individual (175 males and 26 females; with an average age of 57.80 ± 11.8 yr) but due to the poor quality of DNA sample of three cases, we couldnot determine their genotypes so 198 cases were genotyped generally. The genotype frequencies of NF-κB1 -94 ins/del ATTG in healthy individuals of Fars province are shown in Table 2. The percentage of ins/ins homozygote genotype was 46.97%. The frequencies of heterozygote individuals were 42.42% and the percentage of del/del homozygote genotype was 10.61%. Table 3 illustrates a comparison of the NF-κB1 -94 ins/del ATTG genotypes frequencies between different populations.

**Table 2:** Genotype distribution of -94 NF-κB1 ins/del ATTG

***Genotype***	***Frequency No. (%)***	***Gender***	
		***Male***	***Female***
ins/ins	93 (46.97)	81	12
ins/del	84 (42.42)	75	9
del/del	21 (10.61)	18	3
Allele	%	%	
ins	68.18	68.18	
del	31.82	31.82	

**Table 3:** Genotype frequency of -94 ins/del NF-κB1 in different populations

***Population/Genotype***	***II (%)***	***ID (%)***	***DD (%)***	***P-value***
Turkish 1 (20)	46 (46.47)	47 (47.47)	6 (6.06)	ns
Turkish 2 (54)	30	58	12	0.01
Turkish 3 (women) (55)	50 (27)	113 (59)	27 (14)	0.0001
Chinese 1 (56)	43 (21.3)	100 (49.75)	58 (28.85)	<0.0001
Chinese 2 (17)	113 (24)	266 (58)	79 (17)	<0.0001
Chinese 3 (57)	20 (17.24)	62 (53.45)	34 (29.31)	<0.0001
Chinese 4 (58)	97 (24)	183 (45.30)	124 (30.70)	<0.0001
Chinese 5 (59)	44 (30.77)	68 (47.55)	31 (21.68)	0.001
Chinese 6 (women)(60)	135 (36.99)	166 (45.48)	64 (17.53)	0.02
Chinese 7 (61)	379 (34.64)	562 (51.37)	153 (13.98)	0.003
Chinese 8 (62)	81 (15.58)	271 (52.11)	168 (32.31)	< 0.0001
German (63)	118 (38.44)	141 (45.93)	45 (15.63)	ns
Malaysian (64)	16 (6.75)	138 (58.23)	83 (35.02)	< 0.0001
Brazilian (women) (7)	55 (29.1)	88 (46.6)	46 (24.3)	< 0.0001
Chinese-HAN (15)	222 (36.0)	291 (47.2)	103 (16.7)	0.01
Chinese-UYGHUR (15)	147 (41.3)	161 (45.2)	48 (13.5)	ns
Chinese 9 (women) (65)	135 (37.0)	166 (45.5)	64 (17.5)	0.0001
Chinese 10 (14)	166 (31.6)	252 (48.0)	107 (20.4)	0.0001
American non-Jewish (18)	58 (38.9)	70 (47)	21 (14.1)	ns
American Jewish (18)	61 (43)	65 (45.8)	16 (11.3)	ns
Danish (66)	267(34.28)	385 (49.42)	127 (16.30)	0.002
Spanish (67)	31 (31.3)	53 (53.5)	14 (15.2)	0.03
Swedish 1 (68)	116 (26.0)	255 (59.0)	67 (15)	< 0.0001
Swedish 2 (17)	116 (26.0)	256 (58.0)	67 (15)	< 0.0001
Swedish 3 (69)	256(41.16)	270(43.41)	96(15.43)	ns
Southeast Iranian (women) (33)	62(30.5)	106(52.2)	35(17.2)	0.002
Our studied population	93 (46.97)	84 (42.42)	21 (10.61)	

## Discussion

Our results showed the genetic admixture of Iranians with neighboring populations and indicated a gradient of allelic similarity between populations along the Silk Road.

Migration has played a central role in the evolution of human populations. In parallel, moving to a new environment is bound to bring encounters with the new pathogens/allergens/lifestyle. Based on the pronounced role of the immune system in combating pathogens and environmental stressors, the outcome of gene-environment interactions can be reflected in the allelic frequencies of the immune response genes.

**Fig. 2:**
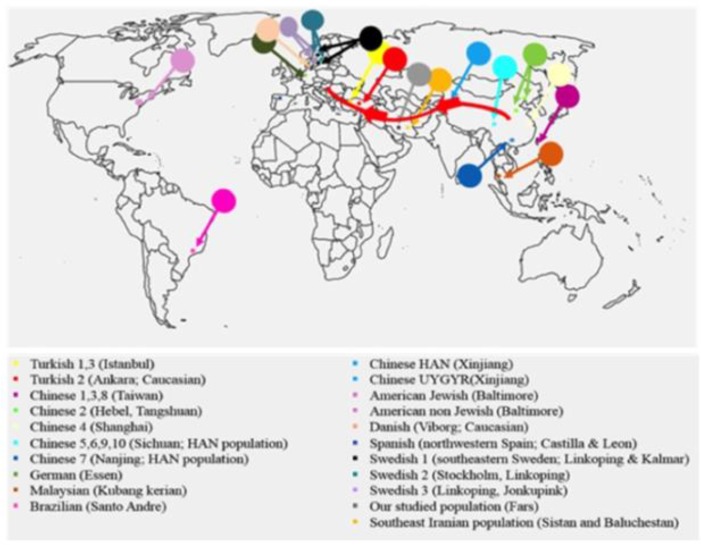
The geographic distribution of populations in which -94 NF-κB1 94 ins/del ATTG polymorphism is reported. The red line represents the silk road

In the human evolutionary history, SNPs placed in autosomal chromosomes, are altered by effects of various factors. Therefore, finding SNP frequencies is a valuable information in terms of connections between different populations (36). Investigating allele frequencies of the immune genes in various populations could be used to track genetics of populations and disease susceptibility (8, 9). In the present study, we investigated the NF-κB1 -94 ins/del ATTG polymorphism in 201 healthy blood donors recruited from Fars blood transfusion center, Shiraz, Fars. The geographic area from the donors recruited was Fars Province in the southwest of Iran, only. We compared genotype and allele frequencies of this SNP in this sample of southwest Iranians with the reported frequencies from different populations of the world (Fig. 2).

**Fig. 3:**
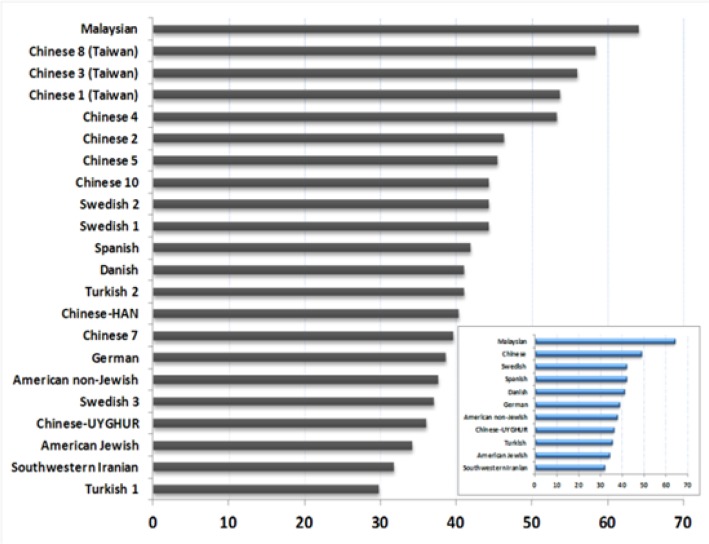
Comparison of the frequency of mutant allele in different human populations

The genotype frequencies of NF-κB1 -94 ins/del ATTG polymorphism were not significantly different between our population and the Chinese-Uyghurs, while they were significantly different from that of Chinese-HAN (Table 3). Uyghurs live in China while they have a Turkic ethnic origin. Archaeological, anthropologic and genetic studies illustrated that Uyghurs genetic makeup is an admixture of Eastern and Western Eurasian populations (37). Therefore, the greater similarity of the frequencies of the NF-κB1 genotypes in Uyghurs than HANs with our FARS population is not unexpected. Moreover, the Uyghurs are Muslims and Muslims generally have similar abstinences and habitsin their lifestyle, affected the evolution of NF-κB1 -94 ins/del genotypes. Many immune-related genes are shown to have been subject to a positive selection by microbial mutualism in recent human history (38, 39). Among the lifestyle-related genes, alcohol dehydrogenase (ADH) variants have been shown to be under selection in eastern Asians in modern era (40). In general, the average frequencies of NF-κB1 -94 ins/del mutant allele (Del) were not different between our population and Turkish populations, however, single studies showed diversity (Fig. 3). The heterogeneity and genetic admixture in Turkish population are well established and previous studies indicated different allele frequencies among the Turkish population due to the heterogeneity (36). We have seen ambiguity in our comparisons with Turkish population. Both differences and similarities of Iranians with Turkish people were reported. Likewise, we observed similarities and differences with Swedish populations which are in line with previous studies (41, 42).

The genotype frequencies of NF-κB1 -94 ins/del ATTG polymorphism did not have a significant difference from that of American Jewish (Ashkenazi) and non-Jewish populations. There are ambiguities in the origin of American Ashkenazi Jewish, but the most accepted theory suggests that their origin is an area, which is currently in Italy (43). Previous studies also have shown a genetic relation between Southwestern Iranians and Italian population while strong evidence for similarities with Ashkenazi Jewish people are lacking (44–47). On the other hand, we observed a genetic similarity based on the genotype frequencies of NF-κB1 -94 ins/del ATTG polymorphism between Germans and our sample of Iranians which is well-founded by the common Aryan ancestry and previous genetic studies (46). Moreover, the phylogenetic trees place Iranians close to German and Italian people (48).

Interestingly, we observed different genotype frequencies of NF-κB1 -94 ins/del ATTGbetween our studied population and other populations such as Spanish and Brazilian, which relates with very few studies on other genes in these populations (49, 50).

The functional significance of NF-κB1 -94 ins/del ATTG polymorphism in susceptibility to different diseases is justified differently. Del/Del genotype that results in decreased expression of p50, affect the p50 homodimer expression more than p50/p65 heterodimer thus reducing p50 homodimer and reducing anti-inflammatory activity. On the other hand, this polymorphism, with reduction of p50 expression, causes heterodimer p50/p65 decline, therefore, leads to diminution of inflammatory activity (18, 20). In either case, NF-κB gene works at the heart of the immune system and directs various immune responses to pathogenic and non-pathogenic stimuli (51). The NF-κB1 -94 ins/del ATTG polymorphism, too, is a functional polymorphism which affects the inflammatory responses thereby shaping the adaptive immune response (6, 18). Such a polymorphism is very likely to be differentially selected under different environmental selective pressures as inter-species evolution is shown to have resulted in the co-evolution of immune genes and pathogens (52, 53).

## Conclusion

Improving our understanding of the global distribution of polymorphisms in immune-related genes developed under selection pressure of pathogenic and environmental encounters has implications for disease association, vaccine design and transplantation studies. The complex nature of immune response demands the study of multiplex analysis of the gene polymorphisms. However, placement of SNPs in the core immune signaling molecules (NFkB, CD1, etc.) along with the well-studied immune genes such as HLA may help to better define the relations between human populations and design population-specific vaccines for pathogens with high rate of variation such as HIV. Lessons from inter-species evolution have taught us that the immune genes and pathogens co-evolve and as we get closer to a more detail-oriented vaccine development strategy, we can benefit from these lessons.

## Ethical considerations

Ethical issues (Including plagiarism, informed consent, misconduct, data fabrication and/or falsification, double publication and/or submission, redundancy, etc.) have been completely observed by the authors.
